# Knowledge of Psychogenic Polydipsia Within Mental Health Services

**DOI:** 10.1192/bjo.2024.135

**Published:** 2024-08-01

**Authors:** Ewelina De Leon, Louise Dunford, Graeme Yorston

**Affiliations:** 1 St Matthews Healthcare, Northampton, United Kingdom; 2 University of Warwick, Coventry, United Kingdom

## Abstract

**Aims:**

Psychogenic polydipsia (PP) is a term used to describe a repetitive behaviour that characterises compulsivity in psychiatric patients resulting in excessive fluid consumption. It is a common clinical problem in patients with severe mental illness, learning disability, autism and acquired brain injury. Up to 20% of patients with schizophrenia have polydipsia, and many develop hyponatraemia and water intoxication, which can lead to irreversible brain damage or death.

Psychogenic polydipsia may not be obvious to staff in a busy care setting, leading to delayed identification and appropriate care.

The objective of this study is to assess the existing knowledge of psychogenic polydipsia among mental health staff and promote greater awareness of the condition.

**Methods:**

To investigate the understanding of psychogenic polydipsia among healthcare staff, an online survey has been chosen as the research method. This survey will help identify any knowledge deficiencies in this area. It consists of both closed and open-ended questions, allowing for quantitative and qualitative analysis. The open-ended questions are designed to provide an opportunity for participants to share their individual experiences. Additionally, the survey will collect information on participants' age groups, years of experience in mental health services, and level of expertise. The survey was created using Qualtrics online survey software. Participant recruitment will be conducted at St Matthews Healthcare, with an estimated sample size of n = 101. The collected data will be analysed using statistical software such as SPSS, NVivo, or other appropriate tools.

**Results:**

The results of this study will be presented. Data are being collected and analysis will be completed in March. The abstract will be updated. These findings will serve as the basis for future recommendations and suggestions.

**Conclusion:**

Comprehending patients' illnesses is a crucial aspect of providing quality healthcare. However, identifying psychogenic polydipsia has proven to be challenging within mental health settings. Failure to recognize excessive fluid intake can result in ineffective treatment, exacerbation of psychiatric symptoms, and in severe cases, coma or even death. The findings of this study have the potential to contribute to the creation of a training program for healthcare providers. Such a program would enable the development of improved care plans for patients who engage in excessive fluid consumption and are at risk of developing hyponatremia and water intoxication.

